# Effects of Deoxynivalenol and Its Acetylated Derivatives on Lipid Metabolism in Human Normal Hepatocytes

**DOI:** 10.3390/toxins16070294

**Published:** 2024-06-26

**Authors:** Zhaoqing Ma, Yuyun He, Yuzhi Li, Qiao Wang, Min Fang, Qing Yang, Zhiyong Gong, Lin Xu

**Affiliations:** 1 College of Food Scienceand Engineering, Wuhan Polytechnic University, Wuhan 430023, China; 2 Key Laboratory for Deep Processing of Major Grain and Oil, Ministry of Education, Wuhan 430023, China; 3 Hubei Key Laboratory for Processing and Transformation of Agricultural Products, Wuhan 430023, China; 4 Key Laboratory of Detection Technology of Focus Chemical Hazards in Animal-Derived Food for State Market Regulation, Wuhan 430075, China

**Keywords:** deoxynivalenol, deoxynivalenol acetylated derivatives, transcriptomics, lipidomics, glycerophospholipid metabolism

## Abstract

Deoxynivalenol (DON), 3-acetyldeoxynivalenol (3-ADON) and 15-acetyldeoxynivalenol (15-ADON) belong to type B trichothecenes that are widely detected in agricultural products as one of the most common classes of mycotoxins. In the present study, we aimed to characterize the alteration of lipid metabolism in normal human hepatocytes by poisoning with DON and its acetylated derivatives. After verifying the hepatotoxicity of the three toxins, DON, 15-ADON, and 3-ADON, the mRNA expression was determined by transcriptomics, and the results showed that DON and 15-ADON had a significant regulatory effect on the transcriptome, in which glycerophospholipid metabolism pathway and phospholipase D signaling pathways have not been reported in studies of DON and its acetylated derivatives. For further validation, we explored lipid metabolism in depth and found that PC (15:0/16:0), PC (16:1/18:3), PC (18:1/22:6), PC (16:0/16:0), PC (16:0/16:1), PC (16:1/18:1), PC (14:0/18:2), PE (14:0/16:0) and PE (18:1/18:3) were downregulated for all nine lipids. Combined with the transcriptome results, we found that hepatic steatosis induced by the three toxins, DON, 15-ADON and 3-ADON, was associated with altered expression of genes related to lipid oxidation, lipogenesis and lipolysis, and their effects on lipid metabolism in L-02 cells were mainly realized through the PC-PE cycle.

## 1. Introduction

Deoxynivalenol (DON), an inhabitant of the B-type trichothecene lineage, finds its recurring presence within an array of cereals. This compound emerges as a product of *Fusarium* within the realm of edibles. It unfolds a comprehensive spectrum of toxicity, extending its grasp over both the human populace and the animal kingdom. Holding a position of prominence within the domain of victuals and provender, DON reigns as a paramount mycotoxin [1,2,3,4]. In 2014, the Food and Agriculture Organization (FAO) of the United Nations, alongside the Joint Expert Committee on Food Additives (JECFA) under the World Health Organization (WHO), recommended that the total intake of DON and its acetylated derivatives should not exceed 1 μg/kg body weight per day [1,5]. A global survey indicated that mycotoxins are present in 80% of agricultural products. Notably, DON is often found with its derivatives, such as 3-acetyl deoxynivalenol (3-ADON) and 15-acetyl deoxynivalenol (15-ADON), which are DON precursors [6]. Both derivatives are highly prevalent in cereals, with contamination levels ranging from 300 to 1000 μg/kg. De Boevre et al. reported detection rates of 77%, 87%, and 73% for DON, 3-ADON, and 15-ADON in cereals and their derivatives, respectively, due to their thermal stability and resistance to processing [7]. Palacios et al. detected DON in all 84 durum wheat samples from Argentina, with a mean level of 1750 μg/kg, and found that 49% were positive for acetylated derivatives of DON [8]. Besides cereals, mycotoxin contamination is also found in vegetables, legumes, fish, and meat, with DON being the most commonly detected mycotoxin in these products, with percentages ranging from 13% in vegetables, 18% in meat, 19% in fish, and 60% in cereals. While 3-ADON was only detected in fish, 15-ADON was present in legumes, vegetables, and fish [9]. Although acetylated derivatives generally have lower detection rates and concentrations compared to DON, they can convert to DON in vivo and in vitro, exhibiting equal or greater toxicity.

There are four metabolic pathways for 3-ADON and 15-ADON in organisms: (1) conversion to DON before entering the gastrointestinal tract; (2) partial conversion within the gastrointestinal tract; (3) complete conversion in the liver; and (4) conversion in the kidneys [10,11,12,13]. These pathways indicate that DON, 3-ADON, and 15-ADON can damage any organ they pass through. The liver, crucial for lipid metabolism and detoxification, is particularly vulnerable. Subchronic and chronic exposure to DON has been shown to affect the liver significantly. Tardivel et al. observed necrosis at the hepatic lobule–portal vein interface in mice exposed to DON (25 μg/kg) for 30 days [14]. Chen et al. noted a reduction in hepatic glycogen, accumulation of haematoxylin granules, thickening of interlobular connective tissue, and loss of smooth endoplasmic reticulum and ribosomes in hepatocytes [15]. Pietsch et al. demonstrated liver injury in pigs and carp caused by DON through enzyme inhibition and oxidative stress [16]. Rats exposed to DON showed increased DNA breaks, elevated lipid peroxidation, and decreased hepatic glutathione [17]. DON-induced hormonal and metabolic dysregulation, as well as various hepatic abnormalities, can lead to non-alcoholic fatty liver disease (NAFLD) in mice [18]. Barbouche et al. observed hepatic steatosis in acutely DON-poisoned mice, linked to changes in the expression of genes involved in lipid oxidation, lipogenesis, and lipolysis. Alassane-Kpembi et al. found that DON downregulated the peroxisome proliferator-activated receptor (PPAR) and liver X receptor-retinoid X receptor (LXR-RXR) signaling pathways, which control lipid metabolism, leading to pro-inflammatory responses and oxidative stress [19]. At the molecular level, DON can significantly induce liver injury by altering the expression of p53, caspase-3, caspase-7, caspase-8, and Bax in various cell lines [20]. Sahu et al. found that exposure to DON (0.1 to 25 μg/mL) significantly reduced mitochondrial function in MH1C1 (rat) cells and decreased the viability of WRL68 (human) cells [5]. Sun et al. observed that DON induces oxidative stress in hepatocytes, affects the expression of ATF3 and p53 (genes related to genome stability), and regulates apoptotic signaling pathways, leading to a high frequency of micronuclei in hepatocytes [21].

The contamination of food by DON and its acetylated derivatives has become a crucial topic in food safety research. Despite extensive studies on DON’s toxicity, especially its acetylated derivatives, most focus on the transcriptomic level. Hence, it is essential to investigate the toxicity of DON and its acetylated derivatives from various perspectives. By analyzing the impact of DON and its derivatives on lipid metabolism in human normal hepatocytes at the transcriptomic and lipidomic levels, we aim to provide a more comprehensive understanding of their toxic effects.

## 2. Results

### 2.1. Effect of DON and Its Acetylated Derivatives on Cellular Activity and Lipid Droplet Accumulation

**Figure 1:**
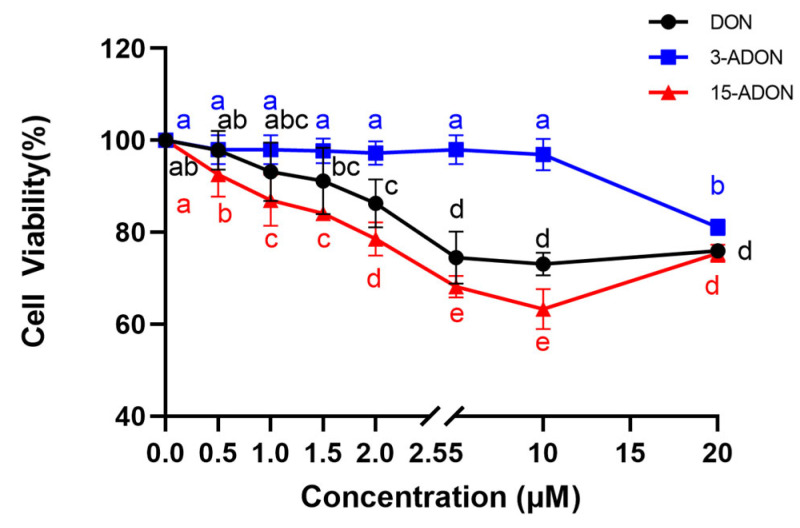
Changes in cellular activity of L-02 cells after 24 h exposure to DON, 3-ADON and 15-ADON environments (n = 5, mean ± SD). Note: Different letters indicate differences between each group after LSD and Duncan two-by-two comparisons at the α = 0.05 level of significance.

**Figure 2:**
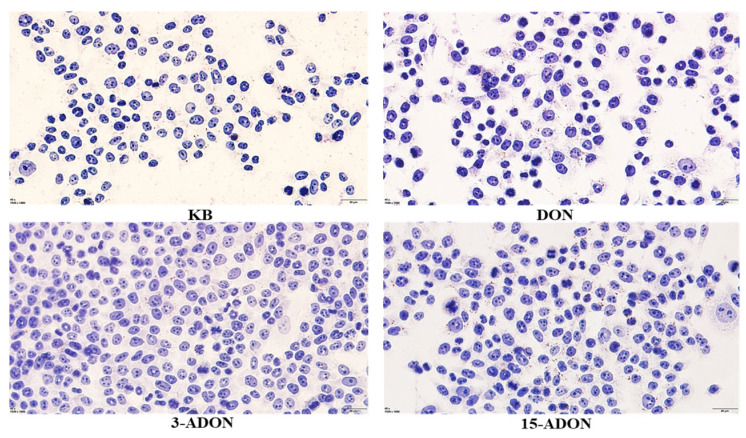
Changes in cellular lipids in L-02 cells exposed to 2 μM DON, 3-ADON, and 15-ADON (400×).

The effects on cellular activities of L-02 cells exposed to various concentrations (0 μM, 0.5 μM, 1 μM, 1.5 μM, 2 μM, 5 μM, 10 μM, 20 μM) of DON, 3-ADON, and 15-ADON for 24 h are depicted in Figure 1. For DON and 15-ADON groups, a dose–response relationship with drug concentration within the 0–10 μM range is observed. Cellular activity decreases as drug concentrations increase. The toxicity rankings of the three toxins on L-02 cells were as follows: 3-ADON < DON < 15-ADON. Considering dietary exposure statistics for the three mycotoxins, it is plausible that the 24 h human intake of DON, 3-ADON, and 15-ADON surpassed 2 μM. At this concentration, the cellular activity of L-02 cells reached 97.72%, 86.33%, and 78.57% respectively, maintaining a survival rate exceeding 80%. Hence, cell samples exposed to 2 μM DON were selected for subsequent experiments. Following a 24 h exposure to 2 μM DON, 3-ADON, and 15-ADON, L-02 cells were stained with Oil Red O Staining to visualize lipid content changes, as presented in Figure 2. Compared with the control group, the lipid production in the 3-ADON group was close to that of the control group, with no significant change; the distribution of lipid droplets was relatively dispersed, whereas the number of lipids in the DON group was significantly increased and the distribution tended to be concentrated. The 15-ADON group was significantly denser, the number of lipids was significantly increased, and the phenomenon of aggregation of lipid droplets could be observed in the 15-ADON group, compared with the remaining three groups. Combined with the results of the cell activity test, it can be seen that the lipid production of L-02 cells was regulated by DON, 3-ADON and 15-ADON, and the degree of regulation was proportional to the degree of the toxicity effect on L-02 cells.

### 2.2. DON and Its Acetylated Derivatives on the Transcriptome Effects

**Figure 3:**
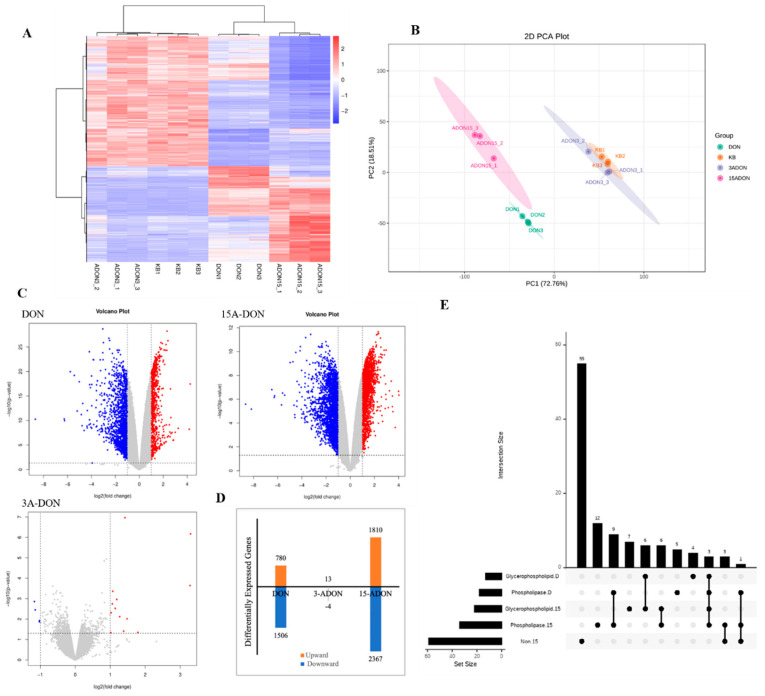
(**A**) Results of mRNA sequencing of L-02 cells; (**B**) plot of principal component analysis of sequencing results; (**C**) comparative volcano plot of differentially expressed genes by exposure group in L-02 cells (Each dot in the figure represents a gene. Blue dots indicate significantly downregulated genes, red dots indicate significantly upregulated genes, and grey dots indicate genes with no significant difference); (**D**) regulation of significantly differentially expressed genes in each group; and (**E**) Venn diagram of differential genes in various lipid metabolism-related pathways.

mRNA was extracted from L-02 cells and subsequently sequenced, with the outcomes visually depicted in Figure 3A,B. Notably, 3-ADON exhibited no significant influence on the transcriptome of L-02 cells, lacking biological significance. In contrast, both DON and 15-ADON distinctly and significantly modulated the L-02 cell transcriptome, as illustrated in Figure 3C. Hence, the forthcoming transcriptome analyses focused exclusively on the DON and 15-ADON groups. As shown in Figure 3D, the incidence of downregulated genes was higher than that of the upregulated genes in the DON and 15-ADON groups. Notably, the degree of gene expression regulation in each exposure group was positively correlated with the corresponding level of cytotoxicity induced by each toxin.

**Figure 4:**
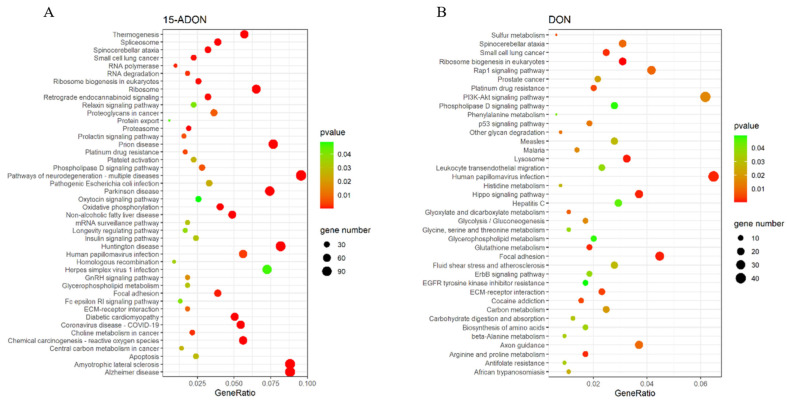
Bubbles of Kyoto Encyclopedia of Gene and Genome (KEGG) pathways of the DEGs. The size of bubbles represents the number of DEGs enriched in the pathway, the colors of bubbles indicate higher enrichment (lower *p*-value) in red: (**A**) KEGG enrichment analysis of significantly differentially expressed genes in the 15-ADON group; and (**B**) KEGG enrichment analysis of significantly differentially expressed genes in the DON group.

**Figure 5:**
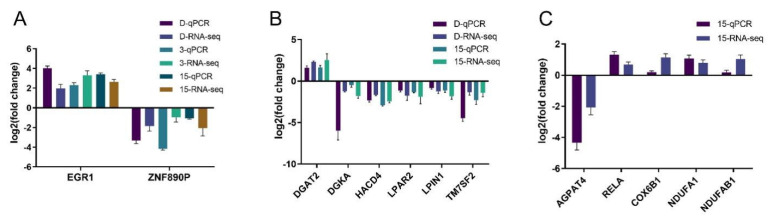
Real-time fluorescence quantitative PCR validates the accuracy of sequencing results: (**A**) qRT-PCR results for EGR1 and ZNF890P in the DON, 3-ADON and 15-ADON groups were consistent with the sequencing results; (**B**) six identical genes in the common regulatory pathway were selected for qRT-PCR in the DON and 15-ADON groups and the results were consistent with the sequencing results; and (**C**) five genes in the nonalcoholic fatty liver pathway, a specific pathway regulated by the 15-ADON group, were selected for qRT-PCR and the results were consistent with the sequencing results.

The KEGG enrichment analysis of differentially expressed genes resulted in pathways with *p*-value < 0.05 being considered significantly regulated, as shown in Figure 4. There were 39 significantly regulated pathways in the DON group, and 44 significant regulatory pathways in the ADON group. Approximately one-third of the total number of regulated pathways attracted our attention, including two pathways related to lipid metabolism that rarely appear in the literature related to DON and 15-ADON, namely, the glycerophospholipid metabolism pathway and the phospholipase D signaling pathway (Table 1). The 15-ADON group had nine overlapping differentially expressed genes in the glycerophospholipid metabolic pathway and the phospholipase D signaling pathway, while the NAFLD pathway was directly associated with the phospholipase D signaling pathway only, with four overlapping differentially expressed genes. The number of differentially expressed genes that overlapped between groups was 9 and 13 for the glycerophospholipid metabolic pathway and phospholipase D signaling pathway, which were co-regulated by DON and 15-ADON, respectively (Figure 3E). The expression of the selected genes was examined by quantitative real-time fluorescence PCR (qRT-PCR), and the results are shown in Figure 5. The results were consistent with the sequencing results and proved that the sequencing results were reliable.

### 2.3. DON and Its Acetylated Derivatives on L-02 Cells Effects of Lipidomics

**Figure 6:**
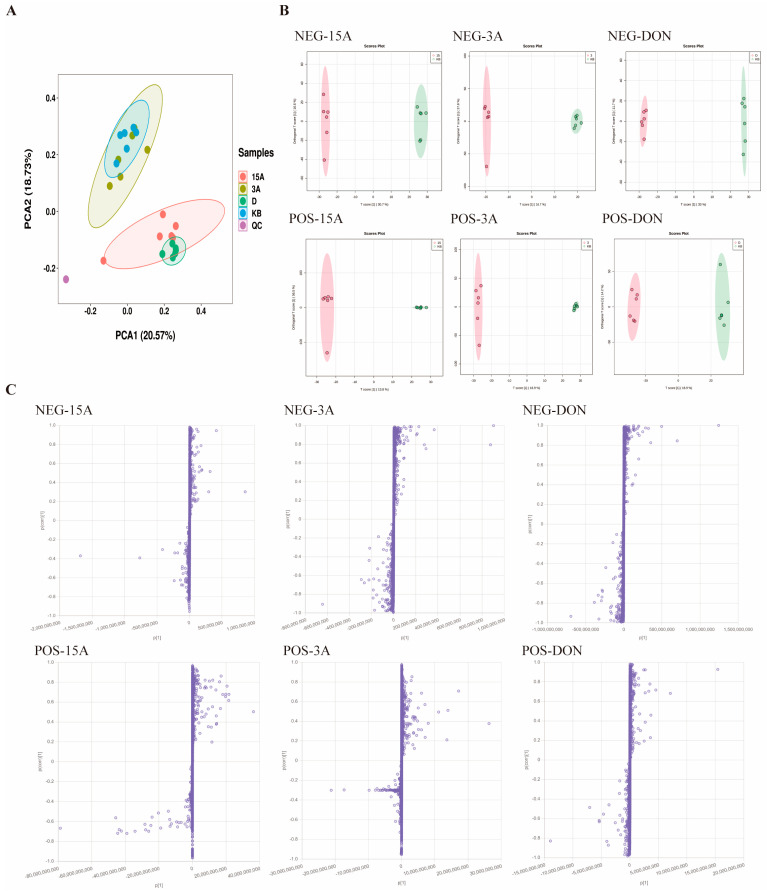
(**A**) PCA scores in the DON, 3-ADON, 15-ADON and control groups; (**B**) plot of OPLS-DA scores in positive and negative ion mode for exposed group vs. control group; and (**C**) S-Plot plot of exposed group vs control group in positive and negative ion mode.

In order to further investigate the abnormalities in lipid metabolism caused by toxins such as DON, 3-ADON and 15-ADON in L-02 cells, we therefore analyzed and studied their metabolites. The data from the Control (KB), QC (QC), DON (D), 3-ADON (3A) and 15-ADON (15A) groups were subjected to PCA analysis; the two-dimensional planes of the results are shown in Figure 6A. The PCA results revealed that metabolites in different groups were separated into clusters. Obvious separation between the control groups and and the DON- and 15-ADON-exposed groups indicated a generated variation in cellular biological systems, leading to a disparity of metabolic profiles and diversity of metabolites. The 3-ADON exposed group did not show clear separation from the control group, which is consistent with our previous findings. The OPLS-DA results for each experimental sample are shown in Figure 6B. The R^2^Y and Q^2^ of the model above 0.4 are acceptable. As shown in Table 2, the model developed in this study satisfies this condition in both positive and negative ion modes, which were within the acceptable range, indicating that the explanatory rate and predictive ability of the model are good and can be used for subsequent analyses. Each dot in the S-plot represents a lipid metabolite in Figure 6C, and the results show that the levels of some of the compounds in each group changed significantly after the intervention, which is represented in the plot by a greater degree of dispersion to the origin of the coordinates, corresponding to a greater weight coefficient of the differential effect of the compounds between the control group and the exposed group. In this study, differential metabolites were screened on the basis of a VIP ≥ 1, an independent samples *t*-test *p*-value < 0.05, combined with the criterion that the multiplicity of change in the peak area of the compound between each group (Fold Change) ≥2 or ≤0.5.

**Figure 7:**
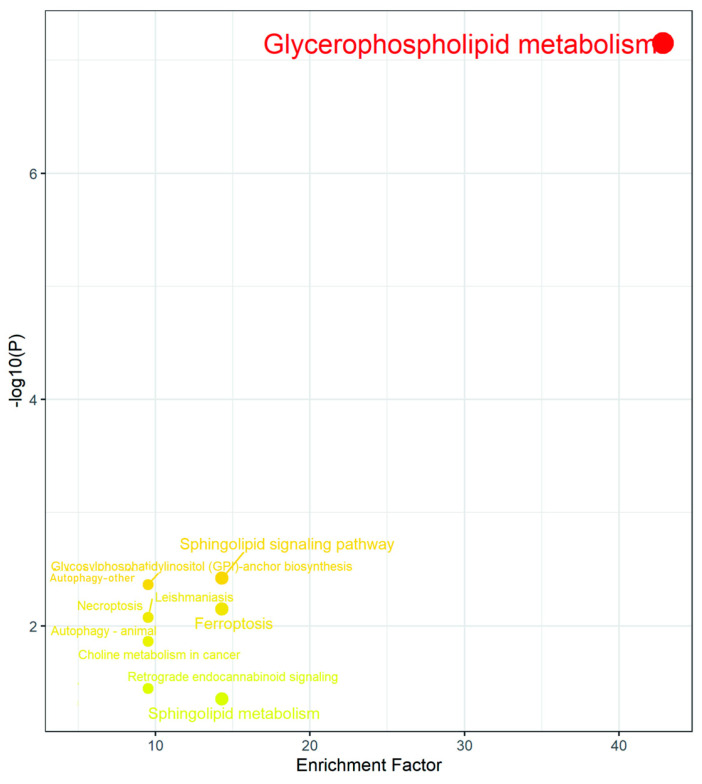
Enrichment map of potential differential gene pathways (Each dot in the graph represents a metabolic pathway; the larger the circle, the further away from the origin, and the darker the colour indicates the greater influence of that metabolic pathway).

The results of the differential metabolites pathway enrichment are shown in Figure 7 and Table 3. The glycerophospholipid metabolism pathway was the most significantly enriched pathway, and this result was consistent with the transcriptome sequencing results. A total of eleven lipids were identified in these pathways, namely, lysophosphatidylethanolamine LPE (C04438), PS (C02737), L-1-lysophosphatidylethanolamine (C05973), cardiolipin (CL, C05980), LPC (C04230), PC (C00157), 1-lysophosphatidylcholine (C04233), PE (C00350), PI (C01194), dihydroceramide (C12126), and sphingomyelin (C00550).

### 2.4. Combined Transcriptomics and Lipidomics Analysis

The results of transcriptomics and untargeted lipidomics showed that the effects of DON, 3-ADON and 15-ADON on the glycerophospholipid metabolic pathway are correlated at the gene and metabolite level; therefore, the glycerophospholipid metabolic pathway will be the main target in this experiment when performing the combined analysis of the two groups. Potential differential metabolites related to glycerophospholipid metabolic pathways were target analyzed and compared with HMDB, Metaboanalyst, KEGG, METLIN and LIPID MAPS databases. Fifteen potential differential metabolites were finally identified (Table 4).

**Figure 8:**
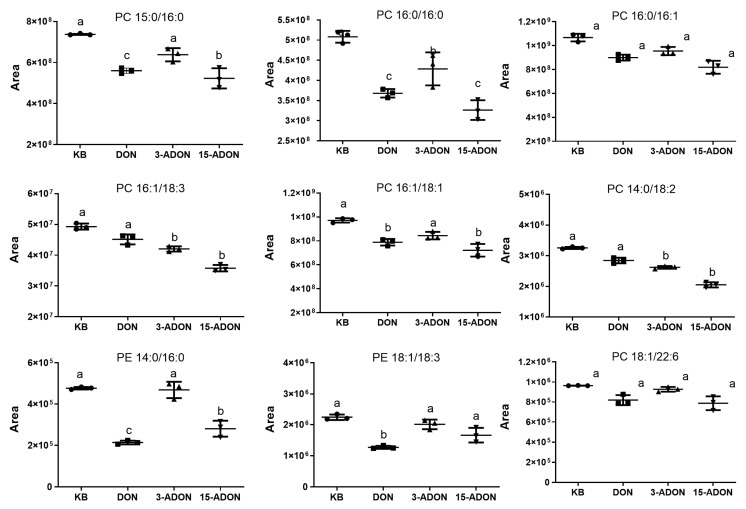
Plot of peak areas of lipid compounds in each exposure group versus the control group (Letters in the graph indicate the level of significance between groups).

Qualitative and semi-quantitative analyses of the 15 potential differential lipid metabolites obtained from the screening were performed using a targeted lipidomics approach. The structures of nine differential metabolites were identified using MRM-IDA-EPI model assays. There are seven PCs: PC (15:0/16:0), PC (16:1/18:3), PC (18:1/22:6), PC (16:0/16:0), PC (16:0/16:1), PC (16:1/18:1), PC (14:0/18:2) and two PEs: PE (14:0/16:0), PE (18:1/18:3). The nine characterized differential metabolites were detected by MRM method and the results are shown in Figure 8. Compared to the control group, the DON, 3-ADON and 15-ADON groups PC (15:0/16:0), PC (16:1/18:3), PC (18:1/22:6), PC (16:0/16:0), PC (16:0/16:1), PC (16:1/18:1), PC (14:0/18:2), PE (14:0/16:0) and PE (18:1/18:3) were all downregulated.

## 3. Disscusion

In a previous study, we investigated the toxic effects of DON, 3-ADON and 15-ADON on colon cancer (Caco-2) cells by means of a transcriptomic approach [22]. Based on the metabolic pathways of these three toxins in the human body, normal human hepatocytes (L-02) were selected in this study to more comprehensively analyze the hepatotoxicity of DON, 3-ADON, and 15-ADON from transcriptomic and lipidomic perspectives. The toxicity of the three toxins was ranked as 15-ADON > DON > 3-ADON. Both 3-ADON (trichothec-9-en-8-one, 3-(acetyloxy)-12,13-epoxy-7,15-dihydroxy-,(3α,7α)) and 15-ADON (trichothec-9-en-8-one, 15-(acetyloxy)-12,13-epoxy-3,7-dihydroxy-,(3α,7α)) have the molecular formula C_17_H_22_O_7_, and they differ in the site of the acetyl group. Desjardins et al. found that C-15 esterification can increase toxicity (from DON to 15-ADON) [23]. The addition of an acetyl group at the R1 position (C3) of the molecule resulted in a loss of potency ranging from 5- to over 100-fold when compared to the parent compound [24]. Oil red O staining showed that DON and 15-ADON significantly promoted the formation of lipid droplets in L-02 cells, whereas 3-ADON had a lesser effect on the formation of lipid droplets in L-02 cells. Therefore, it is reasonable to suspect that the extent of the effect of these three toxins on lipid metabolism in L-02 cells is related to the degree of cytotoxicity.

Transcriptome analysis showed that the transcriptome of L-02 cells was significantly regulated by DON and 15-ADON, whereas 3-ADON did not have a significant effect at the same dose. Notably, 15ADON regulated more genes than DON and was also more toxic than DON at the same dose, again suggesting that toxicity and mRNA alteration are correlated. Pathway enrichment analysis of the differentially expressed genes revealed that the glycerophospholipid metabolic pathway and the phospholipase D signaling pathway were significantly regulated in both sets of results. Both pathways have not been reported in studies of DON and its acetylated derivatives.

**Figure 9:**
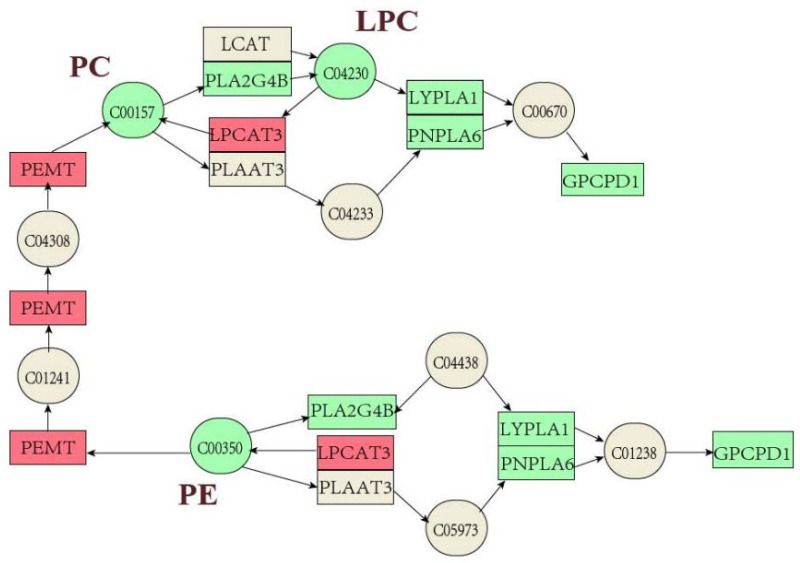
Glycerophospholipid metabolic pathway diagram (green is downregulated, red is upregulated and grey is no significant change).

Lipidomic analysis revealed that the effects of DON, 3-ADON and 15-ADON on L-02 lipid metabolism were mainly mediated through the glycerophospholipid metabolic pathway, sphingolipid signaling pathway, glycosylphosphatidylinositol pathway, autophagy (other) pathway and ferroptosis pathway. The significant modulation of the glycerophospholipid metabolic pathway is consistent with the transcriptomic results. Targeting analysis of potential differential metabolites associated with the glycerophospholipid metabolic pathway resulted in the identification of nine potential differential metabolites, namely: PC (15:0/16:0), PC (16:1/18:3), PC (18:1/22:6), PC (16:0/16:0), PC (16:0/16:1), PC (16:1/18:1), PC (14:0/18:2), PE (14:0/16:0) and PE (18:1/18:3), and all nine lipids were downregulated in the experimental exposure groups. The results of the combined pathway analysis of differentially expressed genes and potentially differential metabolites are shown in Figure 9. PC and PE levels were generally downregulated by PEMT, LPCAT3, LPCAT4, MBOAT1 and MBOAT2, but PC and PE are two larger classes that contain many compounds that are also regulated by various parties; therefore, analysis needs to focus on changes in the content of specific substances. Zhou [25] et al. used metabolomic analysis to study metabolic changes in patients in the early stages of autoimmune hepatitis and detected 14 metabolites as potential biomarkers associated with early stages of liver injury, of which seven glycerophospholipids were downregulated, with the downregulation of LPE (18:0) and LPC (18:0) in line with our findings. Alassane-Kpembi et al. found significant changes in glycerophospholipid metabolic pathways in DON-exposed piglets; the downregulation of PC was consistent with the results of the present assay [26]. Barbouche et al. found that increased expression of genes associated with inflammation and reticuloendoplasmic stress was observed in the liver of DON-exposed mice. Lipid mobilization in adipose tissue (AT) induced by DON intoxication drives hepatic steatosis, as genes encoding lipolytic enzymes are upregulated in AT and plasma concentrations of triglycerides (TG) and non-esterified fatty acids are increased during DON intoxication [18]. This suggests that the downregulation of PC may herald the development of lipid-related diseases and that DON causes hormonal and metabolic dysregulation associated with a range of hepatic abnormalities, leading to nonalcoholic fatty liver disease (NAFLD).

Non-alcoholic fatty liver disease (NAFLD) is a disease characterized by the accumulation of lipids in the liver and is often associated with obesity. NAFLD can progress to non-alcoholic steatohepatitis (NASH), leading to hepatocellular damage (necrosis, fibrosis and inflammation) and cirrhosis, leading to increased morbidity and mortality. The entire spectrum of these liver lesions is known as NAFLD [27]. Glycerophospholipids are important structural components of cell membranes and are involved in multiple cellular signal transduction pathways. Imbalances in glycerophospholipid metabolism may destabilize membranes and promote NAFLD. Yang [28] et al. performed a lipidomic analysis of sera from 100 participants, including 50 NAFLD patients and 50 healthy controls, and found that glycerophospholipid metabolites exhibited significant differences. Wang [29] et al. demonstrated that exposure to bisphenol F leads to non-alcoholic fatty liver disease (NAFLD)-like changes. Notably, the glycerophospholipid metabolic pathway was most pronounced in BPF-induced lipid metabolism disorders. Sun [30] et al. found that L-carnitine significantly improved NAFLD in mice by modulating the glycerophospholipid metabolic pathway. Feng [31] et al. showed that phytosterols ameliorated HFD-induced metabolic abnormalities and NAFLD in mice by modulating the glycerophospholipid metabolic pathway. Increasing evidence suggests that the homeostasis of glycerophospholipids is related to the metabolism of other lipid classes [32]. Thus, the glycerophospholipid metabolic pathway has a pivotal role in lipid metabolism and plays a key role in NAFLD. PC, an abundant and essential glycerophospholipid, is present in the liver and is involved in the structural composition of hepatic cell membranes and in the regulation of important liver functions. There are two major pathways for PC biosynthesis: the cytidine-5-diphosphate choline pathway (CDP-choline pathway) and the phosphatidylethanolamine N-methyltransferase (PEMT) pathway [33]. In this pathway, PE is methylated to PC in three consecutive methylation reactions catalyzed by PEMT. The PC/PE ratio influences numerous liver diseases. Several studies have shown that the relative abundance of both PC and PE is decreased in NAFLD mice, and Arendt [34] et al. showed a lower PC/PE ratio in the liver and erythrocytes of patients with NAFLD compared with healthy controls, which is in agreement with our findings. Therefore, PEMT may play a more important role in balancing the hepatic PC/PE ratio. Jelske et al. found a reduction in the PC/PE ratio from 1.6 to 1.2 in the livers of PEMT^−/−^ mice on a high-fat diet, and the TAG content in the livers of PEMT^−/−^ mice was five times higher than in the livers of wild-type mice [35]. Thus, PEMT may play a more important role in balancing the hepatic PC/PE ratio. PC synthesis is essential for the assembly and secretion of very low density lipoprotein (VLDL) particles, and the absence of PEMT leads to a strong reduction in VLDL secretion. When VLDL is reduced, lipid droplets accumulate in the cytoplasm of hepatocytes, thus causing a fatty liver [36]. A study by Presa et al. found that mice deficient in PEMT were protected from high-fat diet (HFD)-induced obesity and insulin resistance, but developed severe non-alcoholic steatohepatitis when fed HFD [37]. Thus, the upregulation of the 15-ADON group gene PEMT in this experiment may be a protective mechanism in response to lipid droplet aggregation.

In this study, although NAFLD was not among the pathways significantly regulated by the DON group in this study, the phospholipase D signaling pathway is significantly regulated by the DON and 15-ADON groups, and PIK3R2, as a gene co-regulated in both the phospholipase D signaling pathway and the NAFLD pathway, served as a bridge linking the two pathways. class IA phosphatidylinositol 3-kinase (PI3K) is a key mediator of insulin action in the liver, and following insulin stimulation of its receptor, PI3K produces Taniguchi et al. found that deletion of all major regulatory subunits of PI3K in the mouse liver resulted in a significant reduction in PI3K activity and accumulation of PIP3 in the liver following insulin stimulation, with significant defects in glucose and lipid homeostasis as well as liver size and function [38]. Thus, the PI3K pathway is critical for metabolic homeostasis and cell growth.

In addition to PI3K, the peroxisome proliferator-activated receptor (PPAR) is also thought to be critical for NAFLD, with key roles in glucose and lipid metabolism as well as in inflammatory and fibrotic processes. Each isoform of PPAR has its own specific tissue and cell type expression pattern [39,40,41], particularly PPARα. PPARα is predominantly expressed in tissues with high fatty acid oxidation expression and activity in tissues with high rates of fatty acid oxidation, such as the liver (mainly in hepatocytes), and its expression and activity are closely linked to diet. A fat-rich diet increases hepatic PPARα expression in a circadian fashion, and the hypolipidemic effect of PPARα agonists is accentuated when PPARα expression peaks. In addition, PPARα inhibits NASH fibrosis and inflammatory gene expression through protein–protein interactions with the pro-inflammatory transcription factors NF-kB and AP-1 [42]. Thus the downregulation of the PPARA gene in the DON and 15-ADON groups might prove that the hypolipidemic effect of L-02 cells is suppressed.

In addition to this, LPCAT3 that is highly expressed in the liver and intestine catalyzes the production of arachidonic and linoleoyl phosphatidylcholine (PC). LPCAT3 is biased towards LPC and polyunsaturated fatty acyl-CoA (18:2, 20:4, 18:1, 18:3, 22:6) as substrates, and substrate consumption increases with the upregulation of LPCAT3 expression. Furthermore, inhibition of LPCAT3 expression significantly ameliorated UA-induced disruption of lipid metabolism in L-02 cells, with reduced intracellular TC, TG and LDL-C levels [43]. It has, therefore, been hypothesized that small-molecule inhibitors of LPCAT3 may have potential utility in reducing plasma lipid levels in hyperlipidemic individuals [22,44]. In the present experiments, upregulation of LPCAT3 and PEMT did not increase the overall PC content in the liver, probably because upregulation of PEMT reduced the amount of the substrate PE to form PC via the PE methylation pathway, whereas upregulation of LPCAT3 caused polyunsaturated fatty acids in the liver to form polyunsaturated fatty acids PC with LPC to participate in lipid droplet formation, and neutral lipids stored in lipid droplets are hydrolyzed re-esterified and bound to lipoproteins. Thus, the formation of lipoproteins, especially very-low-density lipoproteins, may be the main cause of the overall reduction in PC content [45,46]. This seems to be confirmed by the upregulation of APOO and APOLD1 in the apolipoprotein family in transcriptomic data.

Based on combined transcriptomic and lipidomic analyses, it is clear that genes in the glycerophospholipid metabolic pathway clearly target their corresponding lipid metabolites and that the effects of DON, 3-ADON and 15-ADON on lipid metabolism in L-02 cells are mainly mediated through the PC-PE cycle. These findings contribute to a better understanding of the mechanisms underlying the toxic effects of DON, 3-ADON and 15-ADON, and their impact on lipid metabolism-related diseases.

## 4. Conclusions

In conclusion, our study underscores that lipogenesis in L-02 cells is under the regulatory influence of DON, 3-ADON, and 15-ADON, with their impact correlating to their toxicity levels. Despite distinct variations, all three compounds led to dysregulation of glycerophospholipid metabolism. This disruption, driven by DON, 3-ADON, and 15-ADON through the PC-PE cycle, could potentially instigate lipid metabolism-related disorders, thereby affecting cellular function. Furthermore, our investigation revealed a consistent downregulation of PC and PE content attributed to the collective impact of PEMT, LPCAT3, LPCAT4, MBOAT1, and MBOAT2. These insights offer potential directions for further in vivo experiments, presenting valuable cues for comprehending the mechanisms by which mycotoxins induce alterations in cellular metabolic pathways.

## 5. Materials and Methods

### 5.1. Chemicals

Gibco (Waltham, MA, USA) provided Roswell Park Memorial Institute (RPMI) 1640 medium, trypsin, phosphate buffer salt (PBS), and fetal bovine serum (FBS). Genom (Hangzhou, China) provided the penicillin-streptomycin solution, Servicebio (Wuhan, China) provided the saturated oil red O solution and hematoxylin stain, and the 4% paraformaldehyde fixation and CCK-8 kits were available from Biosharp (Beijing, China). DMSO was obtained from Sigma (St. Louis, MO, USA), and DON, 3-ADON, and 15-ADON standards were obtained from Prebang (Qingdao, China). Chromatography Grade Methanol, Chromatography Grade Methylene Chloride, Chromatography Grade Isopropanol and Chromatography Grade Acetonitrile were supplied by Merck (Shanghai, China). TRizol-Reagent, Ammonium Formate (Chromatography Grade), and Formic Acid (Chromatography Grade) were supplied by ThermoFisher Scientific (Shanghai, China). Cadmium chloride anhydrous was provided by Sinopharm Chemical Reagent (Wuhan, China).

### 5.2. Cell Culture

The China Center for Type Culture Collection provided the normal human hepatocytes (L-02). L-02 cells were cultured in an RPMI 1640 medium containing 10% fetal bovine serum (FBS) and 1% penicillin-streptomycin solution at 37 °C in a humidified (95% humidity) atmosphere containing 5% CO_2_ in a CO_2_ incubator (Memmert, Germany).

The growth of the cells was observed and the cells were passed when they covered about 80% of the bottom of the flask. The cells were washed with PBS buffer solution and then digested by adding trypsin solution containing 0.25% EDTA. After termination of digestion, 1640 complete medium was added, and the cells were divided into two or three T75 flasks, depending on the amount of cells. The incubation was continued in an incubator at 5% CO_2_, 95% humidity and 37 °C, and the liquid was changed after 24 h. The cells were observed for adherence to the wall and passaged after two to three days, according to cell status.

### 5.3. Cytotoxicity Assays

The logarithmically grown L-02 cells were digested and prepared into cell suspension, and then counted with a blood microsphere counting plate. Cells were spread in 96-well plates at a density of 1 × 10^4^ per mL, 100 μL per well, and the side wells were injected with 100 μL of sterile PBS buffer and cultured in an incubator for 24 h. After the cells were attached to the wall, the old medium was aspirated away, and the 1640 complete medium (1640 medium:foetal bovine serum:penicillin-streptomycin = 89:10:1) was added to the blank wells (no cells). In the control group, 100 μL of the 1640 medium was added, and in the experimental group, 100 μL of DON, 3-ADON and 15-ADON at different concentrations (0 μM, 0.5 μM, 1 μM, 1.5 μM, 2 μM, 5 μM, 10 μM, and 20 μM) were added for each single type of toxin exposure in five parallels per group. After 24 h in the incubator, 10 μL of CCK-8 was added to each well, and the incubation was continued for 2–4 h. The OD values of the groups were measured at 450 nm using a microplate reader (PerkinElmer, Waltham, MA, USA) and the cell activity was calculated after repeating the experiment three times.

### 5.4. Oil Red O Staining

L-02 cells grown in logarithmic phase were inoculated in six-well plates at a cell number of 1 × 10^6^ per mL and cultured in an incubator for 24 h. After the cells were attached to the wall, the old medium was carefully aspirated, and 2 μM of DON, 3-ADON, and 15-ADON solutions were added, respectively, with three parallels in each group, and the control group was added with an equal amount of 1640 medium. After 24 h of action, the staining solutions were carefully and gently discarded. After the cells were gently washed with PBS buffer, 4% paraformaldehyde was added and fixed for 30 min or left overnight. After fixation, the cells were washed twice with ultrapure water and soaked in 75% ethanol solution for 10 min. ethanol solution was discarded, and the cells were stained with Oil Red O staining solution (saturated Oil Red O staining solution: deionized water = 3:2, filtered until the solution was clear and free of impurities) for 30 min, the volume of the staining solution covering the bottom of the plate. after 30 min, the cells were decolorized with 75% ethanol solution, and the background color was removed, and then re-stained with hematoxylin for 10 min. After staining, the plate was washed twice with PBS buffer at 30~40 °C, and then observed under a microscope (Olympus, Tokyo, Japan) when the background color turned blue.

### 5.5. RNA Extraction

L-02 cells in the logarithmic phase of growth were inoculated into six-well plates and incubated in the incubator for 24 h to make the cells adherent to the wall, and then 2 μM of DON, 3-ADON, 15-ADON and an equal amount of 1640 medium (control) were added for exposure, and then the solution was removed and rinsed with PBS for 1~2 times after 24 h. Total RNA was extracted from L-02 cells using Trizol reagent (Vazyme, Nanjing, China). The concentration and purity of the RNA samples were examined at 260/280 nm ratio by Nanodrop (Thermo Scientific, Waltham, MA, USA). RNA degradation and contamination were monitored on 1% agarose gel electrophoresis (AGE) by Mini-Sub Cell GT System (Bio-Rad, Minneapolis, MN, USA).

### 5.6. RNA-Seq Analysis

Sequencing of L-02 cell samples exposed to 2 μM DON, 3-ADON and 15-ADON alone for 24 h was performed by Qinda Biotechnology (Wuhan, China). Illumina PE300 library was constructed for sequencing based on Illumina Hiseq 4000 sequencing platform, and the quality of the obtained sequencing data was assessed using FastQC v 0.11.5. Raw data were cleaned using Trimmomatic v 0.36 software to remove splice contamination and reads with a high number (≥10%) of N (N indicates that the base information could not be determined) as well as low-quality sequences (bases with Q ≤ 10 accounted for more than 50% of the overall number of sequence bases). Mapped reads were obtained by sequence alignment of Clean Reads against the reference genome using Hisat2 v 2.2.1 software. DESeq2 analysis and data visualization were performed in R language (R v 4.0.2). Differentially expressed genes (DEGs) were selected using a cut-off threshold, fold change ≥ 2 and adjusted *p*-value ≤ 0.01. The Kyoto Encyclopedia of Genes and Genomes (KEGG) enrichment analyses were carried out using the Profiler software package in R. Pathways considered significant at *p*-value ≤ 0.05, *q*-value ≤ 0.2, *p*-adjustment ≤ 0.01 and *q*-value ≤ 0.05 were selected, respectively [47].

### 5.7. Quantitative Real-Time PCR (qRT-PCR)

To validate the results of differentially expressed genes, DEGs were randomly selected and measured by relative qRT-PCR. Reverse transcription was performed using PrimeScript™ RT Master Mix (Perfect Real Time) (Takara Biomedical Technology, Beijing, China). The cDNA was synthesised according to the known concentration of RNA, based on a standard of 500 ng of RNA per 10 μL of reaction system. the reaction procedure was: 15 min at 37 °C, 5 s at 85 °C, placed at 4 °C, and then entered into the PCR cycle. The software primer 6 was used to design the qPCR primers (Table 5), and then the specificity of the primers was checked with the online BLAST tool on the NCBI website. After amplification, nucleic acid gel electrophoresis was performed to test the specificity and amplification efficiency of the amplification products.

TB Green Kit (Takara) was used for qRT-PCR. The temperature conditions were as follows The ß-actin gene was used as the reference gene. The operation was performed according to the instructions of TB Green Premix Ex Taq^TM^ II. kit (Takara). The reaction system was TB Green Premix Ex Taq^TM^ II. 12 μL, 0.5 μL of each upstream and downstream primer, 2 μL of cDNA, and 9.5 μL of RNase-Free H_2_O. The reaction procedure was as follows: 95 °C for 30 s, 95 °C for 5 s, and 60 °C for 30 s for 40 cycles. The melting curves were plotted at 95 °C for 10 s, 60 °C for 5 s, and 95 °C for 5 s. The primer sequences used for qRT-PCR are listed in Table 1. Three biological replicates were set up for all gene expression analyses, and the relative gene expression levels were calculated using the 2^−ΔΔCt^ method [48].

### 5.8. Metabolomic Analysis

L-02 cells in the logarithmic phase of growth were digested and prepared into cell suspension, and the number of cells at a concentration of 1 × 10^7^ cells/mL was spread on a petri dish and cultured in an incubator for 24 h. After the cells were adhered to the wall, the old medium was gently aspirated, and 2 μM of DON, 3-ADON, and 15-ADON solutions were added, respectively, and the control group was added with an equal amount of 1640 medium, with 6 parallels in each group. After 24 h, the solution was discarded, the cells were washed with PBS buffer, trypsin digestion was added, and the cell suspension was collected and centrifuged at 1200 rpm for 5 min. The cells were washed with pre-cooled PBS and centrifuged for two times, and before the last centrifugation, the cell suspension was transferred to a 2 mL EP tube. After centrifugation, all the supernatant was aspirated, and then the EP tube was inactivated quickly by placing it in liquid nitrogen and then into the refrigerator at −80 °C to be tested.

Each cell sample was ground for 9 min in a high-throughput tissue grinder (Scientz-192, Ningbo, China) with 2 grinding beads and 0.6 mL methanol-dichloromethane solution (2:1 *v*/*v*). Following that, 0.6 mL methanol-dichloromethane solution (2:1 *v*/*v*) and 0.5 mL ultra-pure water were added. The system was vortexed until uniform and centrifuged (TGL-16M, Shanghai, China) at 4 °C and 13,000 rpm. A total of 0.5 mL of methylene chloride solution was added to the mixture, which was vortexed and centrifuged for 10 min after absorbing the upper aqueous phase solution into tube A and the lower organic phase solution into tube B. The organic phase solution was placed in the lower layer of the two tubes in centrifuge tube C and dried with the nitrogen blower (MTN-5800, Tianjin, China). After drying, the samples were treated with a 1 mL isopropyl alcohol-acetonitrile (9:1 *v*/*v*) solution for resolution. After resolution, the samples were centrifuged for 10 min at 4 °C and 13,000 rpm. The supernatant was aspirated into the injection bottle and testing machine, then pipetted into the injection vial and for the assay. A total of 15 μL of each of the ground samples was placed into a 2 mL centrifuge tube and vortexed to mix thoroughly. The above procedure was repeated and 1 mL of the supernatant was placed into the injection bottle as a quality-control sample. Six parallels were set up for each group.

For the metabolomic analysis, an ultra-high-performance liquid chromatography-tandem high-resolution mass spectrometry platform (Vanquish UHPLC-Qtrap 6500+ system, Thermo Scientific, Shanghai, China) was used.

### 5.9. Statistical Analysis

Significance was analyzed using IBM SPSS Statistics 21, *p* < 0.05, and the sequencing results were enriched and analyzed using the R language. MS-DIAL software was used for peak filtering, peak identification, peak alignment, normalization, standardization and compound identification of the raw data. Metabo Analyst 5.0 was used for multivariate statistical analysis and metabolic pathway analysis of the normalized data.

## Figures and Tables

**Table 1:** Lipid metabolism-related pathways in KEGG results.

	ID	Pathway	Gene
DON	has 00564	Glycerophospholipid metabolism	*LPCAT3*/*MBOAT2*/*PNPLA6*/*DGKA*/*LPIN1*/*PLD3*/*PLA2G15*/*DGKQ*/*ETNK2*/*PLD2*/*LPCAT4*/*PNPLA7*/*PLD4*
	has 04072	Phospholipase D signaling pathway	*PLCB2*/*RALGDS*/*PLCB3*/*DGKA*/*PDGFB*/*CYTH2*/*PLCG1*/*DGKQ*/*LPAR5*/*PLD2*/*LPAR2*/*PTK2B*/*PIK3R2*/*DNM3*/*RAPGEF4*/*PIK3CD*/*SHC3*/*PDGFRB*
15-ADON	has 04932	Non-alcoholic fatty liver disease	*SDHB*/*NDUFS5*/*JUN*/*AKT3*/*NDUFB8*/*CYP2E1*/*NDUFS3*/*COX8A*/*RELA*/*NDUFS8*/*NDUFC2-KCTD14*/*NDUFC2*/*TNFRSF1A*/*NDUFA4L2*/*NDUFA12*/*COX6A1*/*EIF2S1*/*FOS*/*NDUFB1*/*NDUFB10*/*NDUFAB1*/*COX4I1*/*SOCS3*/*NDUFV2*/*UQCR11*/*NDUFA11*/*NDUFA7*/*NDUFB7*/*PIK3R2*/*NDUFA13*/*UQCRFS1*/*COX6B1*/*GSK3A*/*NDUFA3*/*COX5B*/*NDUFB3*/*IRS1*/*UQCR10*/*NDUFA6*/*PPARA*/*NDUFB4*/*PIK3CA*/*NDUFS6*/*NDUFS4*/*PIK3R1*/*COX7C*/*UQCRQ*/*NDUFA2*/*MAPK13*/*CYCS*/*MLXIPL*/*NDUFB2*/*COX6C*/*NDUFB9*/*CYC1*/*NDUFA8*/*NDUFB11*/*COX7B*/*NDUFA1*
	has 04072	Phospholipase D signaling pathway	*DNM3*/*ARF1*/*AKT3*/*HRAS*/*GAB2*/*DGKA*/*KITLG*/*DGKH*/*SOS2*/*PLCB2*/*JMJD7-PLA2G4B*/*PLA2G4B*/*PLD2*/*GRB2*/*PIK3R2*/*LPAR2*/*CYTH2*/*SOS1*/*RAPGEF4*/*PLCB1*/*PLCG1*/*PLD1*/*PIK3CA*/*DGKQ*/*PIK3R1*/*F2R*/*PDGFRB*/*AGPAT4*/*CYTH3*/*DGKI*/*PTK2B*/*SHC3*/*TSC1*/*RALGDS*
	has 00564	Glycerophospholipid metabolism	*LPCAT3*/*GPD1*/*DGKA*/*DGKH*/*PLD4*/*LPCAT4*/*JMJD7-PLA2G4B*/*PLA 2G4B*/*PLD2*/*PEMT*/*LPIN2*/*MBOAT2*/*LPIN1*/*GPCPD1*/*PLA2G6*/*CHKB*/*PLD1*/*DGKQ*/*MBOAT1*/*AGPAT4*/*DGKI*/*PNPLA7*

**Table 2:** Exposure group vs. control group cell samples OPLS-DA model evaluation parameters.

Ion Mode	Group	R^2^Y	Q^2^
Postive	DON	0.967	0.704
	3-ADON	0.808	0.550
	15-ADON	0.513	0.501
Negtive	DON	0.989	0.928
	3-ADON	0.825	0.557
	15-ADON	0.986	0.927

**Table 3:** Enrichment results for potential differential gene KEGG pathways (top five *p*-values).

Pathway Name	*p*-Value	Adj. *p*-Value
Glycerophospholipid metabolism	3.22 × 10^−7^	8.7 × 10^−6^
Sphingolipid signaling pathway	0.004887	0.033762
Glycosylphosphatidylinositol (GPI)	0.005136	0.033762
Autophagy—other	0.005136	0.033762
Ferroptosis	0.009069	0.033762

**Table 4:** Potential differential lipid metabolites.

Compound Number		Summation Ions	Compound Name	*p*-Value	15A	3A	DON
LIPIDMAPS	HMDB				log2(FC)	log2(FC)	log2(FC)
LMGP01010566	HMDB0007969	[M + HCOO]^−^	PC 16:0/16:1	3.31 × 10^−7^	−1.31953	−0.25847	−1.62121
LMGP01010535	HMDB0007936	[M + HCOO]^−^	PC 15:0/16:1	3.40 × 10^−5^	−1.38383	−0.40766	−1.25755
LMGP01011485	HMDB0008008	[M + HCOO]^−^	PC 16:1/18:3	3.46 × 10^−5^	−1.58501	−0.0225	−1.98641
LMGP01010688	HMDB0008005	[M + HCOO]^−^	PC 16:1/18:1	5.38 × 10^−5^	−0.83986	−0.00508	−2.15982
LMGP01010564	HMDB0000564	[M + H]^+^	PC 16:0/16:0	5.79 × 10^−5^	−1.23797	−0.52877	−1.17285
LMGP02010302	HMDB0008824	[M − H]^−^	PE 14:0/16:0	0.000104	−1.02936	−0.0167	−1.03175
LMGP01010492	HMDB0007873	[M + H]^+^	PC 14:0/18:1	0.000106	−1.53803	−0.27126	−1.1184
LMGP01010690	HMDB0008006	[M + H]^+^	PC 16:1/18:2	0.000203	−1.15744	−0.00891	−1.4226
LMGP01010496	HMDB0007874	[M + HCOO]^−^	PC 14:0/18:2	0.000456	−0.68962	−0.39123	−0.53687
LMGP01010496	HMDB0007874	[M + HCOO]^−^	PC 16:1/16:1	0.002201	−0.68962	−0.39123	−0.53687
LMGP01010913	HMDB0008123	[M + H]^+^	PC 18:1/22:6	0.009175	−0.75511	−0.31468	−0.50407
LMGP01012166	HMDB0008118	[M + H]^+^	PC 18:1/22:1	0.009942	−0.90162	−0.22761	−0.6374
LMGP01010535	HMDB0007936	[M + H]^+^	PC 15:0/16:1	0.02051	−0.74051	−0.26041	−0.20947
LMGP01010532	HMDB0007935	[M + H]^+^	PC 15:0/16:0	0.022227	−0.62339	−0.18916	−0.31691
LMGP02011197	HMDB0009062	[M + H]^+^	PE 18:1/18:3	0.026896	−0.55402	−0.01663	−0.29485

**Table 5:** Gene primer sequences.

Primer Name	F	R
*ß-actin*	CCTTCCTGGGCATGGAGTC	TGATCTTCATTGTGCTGGGTG
*AGPAT4*	GCTTTGGGCTGTTAGGGGGCTC	AGGAAAAAATACTTCTCGGGGT
*DGAT2*	GAACCGCAAGGGCTTTGTGAAA	GCATGGGGCGAAACCAATGTAT
*DGKA*	ATGTTCCTGATAGCCGGATTTT	AAGGGGACTGGGTCACTTTTTT
*HACD4*	CTTTGCCAATCCGTATCTCTCC	CCCTCTTCAACTTTGCCTCTTC
*LPAR2*	GCCACCCCCGCTACCGAGAGAC	CAAGAGTACACAGCAGCATTGA
*TM7SF2*	GGCAGGAATTGAAGGACAAGAG	CGAAGAAACAGATACGAGGGTT
*RELA*	AGAAGAGCAGCGTGGGGACTAC	TGCACATCAGCTTGCGAAAAGG
*COX6B1*	GCTTCCCCAACCAGAACCAGAC	TCAGCCCGTTGCTCATCCCAGT
*NDUFA1*	ATGTGGTTCGAGATTCTCCCCG	TCTTTCCATCAGACTCCAGTGA
*NDUFAB1*	CCTCTCAGCACCGCTCTCTGCT	TCCTGGATGCCCTCTAACGTCA
*EGR1*	TCTGACCCGTTCGGATCCTTTC	CTCCACCAGCACCTTCTCGTTG
*ZNF890P*	CAGGGGTCACTGTCATTCGGGG	TTCGTGTTGCTGTTGGCTATCT

## Data Availability

The data presented in this study are available on request from the corresponding author due to project requirements.
